# They *played* with the *trade*: MEG investigation of the processing of past tense verbs and their phonological twins

**DOI:** 10.1016/j.neuropsychologia.2012.10.019

**Published:** 2012-12

**Authors:** Rachel Holland, Lisa Brindley, Yury Shtyrov, Friedemann Pulvermüller, Karalyn Patterson

**Affiliations:** a Institute of Cognitive Neuroscience, University College London, 17 Queen Square, London WC1N 3AR, UK; b Cardiff University Brain Research Imaging Centre, Cardiff, UK; c Free University of Berlin, Department of Philosophy, Habelschwerdter Allee 45, 14195 Berlin, Germany; d Department of Clinical Neurosciences, University of Cambridge, Cambridge, UK; e MRC Cognition and Brain Sciences Unit, Cambridge, UK

**Keywords:** Past tense inflection, Magnetoencephalography, Phonology, Morphology, Mismatch negativity

## Abstract

How regular and irregular verbs are processed remains a matter of debate. Some English-speaking patients with nonfluent aphasia are especially impaired on regular past-tense forms like played, whether the task requires production, comprehension or even the judgement that “play” and “played” sound different. Within a dual-mechanism account of inflectional morphology, these deficits reflect disruption to the rule-based process that adds (or strips) the suffix -ed to regular verb stems; but the fact that the patients are also impaired at detecting the difference between word pairs like “tray” and “trade” (the latter being a phonological but not a morphological twin to “played”) suggests an important role for phonological characteristics of the regular past tense. The present study examined MEG brain responses in healthy participants evoked by spoken regular past-tense forms and phonological twin words (plus twin pseudowords and a non-speech control) presented in a passive oddball paradigm. Deviant forms (played, trade, kwade/kwayed) relative to their standards (play, tray, kway) elicited a pronounced neuromagnetic response at approximately 130 ms after the onset of the affix; this response was maximal at sensors over temporal areas of both hemispheres but stronger on the left, especially for played and kwayed. Relative to the same standards, a different set of deviants ending in /t/―—plate, trait and kwate—―produced stronger difference responses especially over the right hemisphere. Results are discussed with regard to dual- and single-mechanism theories of past tense processing and the need to consider neurobiological evidence in attempts to understand inflectional morphology.

## Highlights

► MEG-evoked mismatch negativity to inflected past-tense forms. ► Pronounced neuromagnetic responses to deviants elicited 130 ms after onset of affix. ► Maximal responses localised to left hemisphere temporal sensors. ► Voiceless ‘affixes’ (-t rather than -d) elicited stronger right hemisphere response.

## 1 Introduction

In everyday conversation, much of what we say refers to past events. For example, “I played chess until 1 am and then slept late this morning”. Despite the seeming ordinariness of this phenomenon, the procedures by which we produce and comprehend the past-tense forms of the verbs in our vocabulary are much debated in cognitive science and neuroscience. The majority of English verbs are so-called regular because they form their past tenses via a consistent transformation to the stem: the morpheme *-ed* is always added to the orthographic form (e.g., played, pressed, planted) and is realised as one of those three allophones (/d/, /t/, or /id/) in speech, depending on the phonetic characteristics of the final phoneme of the stem. There are, however, exceptions to this typical pattern: approximately 180 monomorphemic irregular verbs form their past tenses in a variety of other ways (e.g., sleep–slept, hit–hit, run–ran, lend–lent, think–thought).

Opinions differ as to whether these descriptive differences between regular and irregular verbs are reflected in genuine differences in mental representation and process. The dual-mechanism account, as articulated by Pinker, (1991), (1998), Pinker & Ullman (2002), argues for two separate processes of verb inflection, each specialised for one of the verb classes. The regular past tense is generated in speech production by a rule-based process that adds the suffix ‘-*ed*’ to any stem that does not have an irregular form listed in the lexicon. The process is argued to be automatic and obligatory, and thus not affected by non-grammatical characteristics of the stem. Irregular past-tense forms, by contrast, are considered to be stored alongside other lexical entries; the presence of such a form blocks application of the ‘add -*ed*’ rule. Tyler and Marslen-Wilson’s explanation of past tense comprehension (e.g., Tyler et al., 2002b; Tyler, Randall, & Marslen-Wilson, 2002c) shares the same general approach of two separate mechanisms: regular past-tense forms are processed by a dedicated morpho-phonological parsing mechanism which strips the affix to allow access to the lexical representation of the stem, whereas irregular past tenses rely upon a separate full-form route.

An alternative to dual-mechanism accounts suggests that both regular and irregular past tense forms are computed within a single, distributed system based on mapping relationships between form and meaning. On this view, all verbs activate phonological and semantic representations in the service of generating the past tense, but regular and irregular verbs place differential emphasis on these two sources of information (Joanisse & Seidenberg, 1999; McClelland & Patterson, 2002; Rumelhart & McClelland, 1986). Because of the overwhelming consistency in the regular past-tense ending, the process of inflecting these verbs does not require much in the way of word-specific knowledge of the kind that would accompany semantic processing. On the other hand, regular verbs stress phonological processes (a) because the past-tense form always has more phonemes than the stem, and (b) because a number of regular past-tense forms (such as ‘loved’ or ‘trimmed’) follow an atypical phonological pattern for English: no monomorphemic words end in combinations like /vd/ or /md/ (Burzio, 2002). In contrast, the great majority of irregular past tense forms are phonologically simple and have a spoken length that, relative to their stems, is either equivalent (run–ran: 3 to 3 phonemes) or even shorter (stand–stood: 5 to 4 phonemes). By virtue of having such word-specific, unpredictable past-tense forms, however, irregular verbs are not helped by – indeed suffer interference from – the overwhelming majority of regular verbs. Irregular past-tense forms, especially less frequent ones that are not constantly being produced, therefore require additional word-specific knowledge, and this can be provided by semantic processing. Within the single-mechanism account, the apparent dichotomy between regular and irregular verbs arises as an emergent property of the graded mappings between form and meaning, rather than representing a predetermined, categorical distinction.

Differences between the two verb classes have been observed in data from developmental (Kuczaj, 1977), behavioural (Gonnerman, Seidenberg, & Andersen, 2007; Kielar, Joanisse, & Hare, 2008), neuroimaging (Beretta et al., 2003; Desai, Conant, Waldron, & Binder, 2006; Jaeger et al., 1996; Joanisse & Seidenberg, 2005; Marslen-Wilson & Tyler, 2007; Oh, Tan, Ng, Berne, & Graham, 2011; Tyler, Stamatakis, Post, Randall, & Marslen-Wilson, 2005), electrophysiological (Gross, Say, Kleingers, Münte, & Clahsen, 1998; Münte, Say, Clahsen, Schlitz, & Kutas, 1999; Penke et al., 1997; Rodriguez-Fornells, Münte, & Clahsen, 2002; Weyerts, Penke, Dohrn, Clahsen, & Münte, 1997) and neuropsychological studies (Bozic, Marslen-Wilson, Stamatakis, Davis, & Tyler, 2007; Marslen-Wilson & Tyler, 1997; Patterson, Lambon Ralph, Hodges, & McClelland, 2001; Tyler et al., 2002a; Tyler et al., 2002c; but see Faroqi-Shah, 2007). Dissociations observed in neuropsychological studies have been used to argue for separate, isolable systems that can be damaged independently of one another—as per the longstanding tradition in cognitive neuropsychology (Shallice, 1988). Double dissociations, however, do not require separate modular processors and corresponding separate brain regions, but can also be explained by a distributed account assuming differential distributions of the relevant processes (Plaut, 1995; Pulvermüller & Preissl, 1991). Furthermore, at least in the realm of verb processing, the supposedly ‘preserved’ class often yields reduced performance relative to controls. For example, in the screening experiment of Bird, Lambon Ralph, Seidenberg, McClelland and Patterson (2003), patients with post-stroke nonfluent aphasia made significantly more errors on regular than irregular past-tense forms (i.e., an irregular>regular “dissociation”); but the patients’ success on the irregular forms was also *substantially* below normal. In fact, most of the existing data on this topic can be largely accounted for by any of the theoretical positions on the table, which is presumably why none has yet dropped off that table.

In the past tense debate, the link between cognitive theory and neurobiological mechanisms has sometimes been ignored. One neurobiological approach to the problem views meaningful word stems as biologically distinct from the grammatical affixes that the stems carry (Pulvermüller, 1995, 2003). The stems of nouns and verbs refer to objects and actions whose meanings are composed of multiple and varied sensory and motor features. According to this view, such stems are therefore neurally represented as distributed cortical systems linking the form of the word – conceptualised as a circuit in left-perisylvian language cortex – to more widely distributed networks reaching into multimodal, sensory and motor areas of both cortical hemispheres. Grammatical affixes, on the other hand, largely lack referential-semantic links; their neural representations may therefore be confined to left-lateralised perisylvian space. This model predicts that both verb stems and past-tense forms like *drank* or *thought* (with no explicit affixes) should elicit relatively bilateral distributed brain responses, whereas responses to inflectional affixes should be left-lateralised. Note that these contrasting patterns of laterality can also be interpreted as a reflection of differential semantic and phonological processing, because semantic knowledge is certainly bilaterally represented whereas phonology is probably a specialised function of the left hemisphere (Lambon Ralph, McClelland, Patterson, Galton, & Hodges, 2001; Pulvermüller, 1999). The differential laterality hypothesis for stems and affixes is thus consistent with both single and dual mechanism accounts.

One experimental paradigm that has played a significant role in the neuropsychological component of this debate is an auditory same-different judgement task in which patients are asked to judge whether two spoken words are the same or different. Critical ‘different’ trials consist of (a) pairs composed of the stems and inflected forms of regular verbs, e.g., “play–played” or “press–pressed” and (b) word pairs which share the same phonological relationship as in the former pairs but lack any true morphological relationship, e.g., “tray–trade” or “chess–chest”. We refer to these latter as phonological twins. Dual-mechanism models that place a strict emphasis on morphological processing predict distinctly different patterns of response to these two conditions, whereas single-mechanism accounts, emphasising phonological processing, predict that the two conditions will yield similar outcomes. Available neuropsychological data, from patients with nonfluent aphasia following left-hemisphere stroke, are thus far equivocal: two studies have reported accuracy of performance as largely the same in the two conditions (Bird et al., 2003; Tyler et al., 2002c) whereas the latter study, which also measured the patients’ reaction times, revealed significantly slower ‘different’ RTs to pairs including real inflections than to phonological-twin pairs. The hypothesis regarding differential laterality for stems and affixes also predicts a significant difference between true inflected words and phonological twins, but clearly this outcome can only be assessed with a technique that provides information about patterns of brain activity. That is the purpose of the study reported here.

One further aspect of Bird et al. (2003) study requires mention here as it formed the basis of one of the main issues addressed by the current experiment. As well as ‘different’ pairs with regular past-tense forms (such as “play–played”) and phonological twins (such as “tray–trade”), additional ‘different’ stimuli in that neuropsychological same-different judgement study consisted of pairs like “play–plate”. The purpose of this type of stimulus pair was to investigate further the hypothesis that phonological factors are strongly implicated in the difficulty shown by nonfluent aphasic patients in comprehending and producing past-tense regular forms. “plate”, of course, is not a verb; but phonologically speaking, it resembles “played” in consisting of the ‘stem’ “play” followed by an alveolar, though /t/ instead of /d/. There is, however, an additional difference between “played” and “plate”: in “played”, as in all regular past-tense forms in English, the terminal phoneme of the word is consistent in voicing with the preceding phoneme, which is the final phoneme of the verb stem. In “plate”, on the other hand, the last two phonemes are discrepant in voicing: the vowel is voiced but the /t/ is not. One hypothesis in Bird et al. (2003) study was that this voicing contrast might make it easier for the phonologically/phonetically impaired aphasic patients to hear pairs like “play–plate” or “he–heat” as different words than pairs like “play–played” or “he–heed”. This hypothesis was supported by the results: independent of morphological status, the patients were considerably more successful at making correct ‘different’ judgements to pairs containing the voicing discrepancy. A similar manipulation was included in the current MEG experiment to determine whether the brains of healthy participants would also be sensitive to this phonetic factor.

The current study employed magnetoencephalography (MEG) to characterise the pattern of brain responses of healthy participants to spoken stimuli in a passive oddball paradigm. In such a paradigm, a mismatch component of the auditory event-related potential can be elicited by any detectable change (deviant) in a stream of regular (standard) auditory events. Such passive mismatch responses have proven to be a sensitive tool for probing automatic neural discrimination of phonemes, words and inflectional affixes (Pulvermüller & Shtyrov, 2006; Shtyrov & Pulvermüller, 2002). Furthermore, this paradigm is conceptually similar to the same-different task just described, with a salient difference being that it can address the early, automatic neurophysiological process of change detection, rather than requiring overt responses from the participant. The experiment included four different conditions defined by the identity of the standard stimulus: the regular verb play; the real-word non-verb phonological twin tray; the pseudoword phonological twin kway; and as a control condition, an unintelligible mixture of the other three standards. In each condition, there were two forms of deviant. One form of deviant consisted of the standard stimulus appended with a /d/ ending: for the three word and pseudoword conditions, this resulted in played, trade and kwade (or kwayed). The second form of deviant consisted of the standard stimulus appended with a /t/ ending: for the three word and pseudoword conditions, this resulted in plate, trait and kwate.

The study was designed to evaluate three major issues. First, as predicted by the neurobiological theory described above: would there be a different pattern of early neuromagnetic responses to affixed words and pseudowords compared to monomorphemic words and unintelligible stimuli? Second, with different predictions coming from dual- vs. single-mechanism accounts of past-tense verb processing: would the pattern of brain responses to trade as a deviant to tray be similar to or different from the responses to played as a deviant to play? Finally, would the patterns of brain response differ to deviants ending in /d/ vs. /t/?

## 2 Methods

### 2.1 Subjects

Eighteen healthy right-handed (handedness tested according to Oldfield (1971)) native speakers of British English (age 20–32 years, 6 males) participated in the experiment. All had normal hearing and no previous history of implants, seizures, neurological or psychiatric disease.

### 2.2 Stimuli

**Fig. 1:**
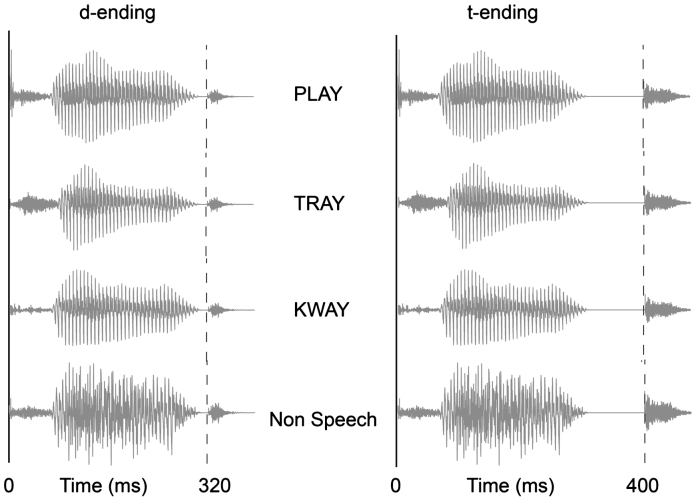
Waveforms of word, word-like and non-speech stimuli used in the four conditions of the experiment. In each condition, deviant and standard stimuli only differ in their endings.

The experimental stimulus set consisted of four different conditions. Each condition comprised a single, frequently presented standard stimulus and two much less commonly occurring deviant stimuli that differed from the standard by the presence of an additional /d/ or /t/ ending (Fig. 1, Table 1). Two main conditions used real spoken words for standards and deviants: play–played–plate and tray–trade–trait. One control condition, consisting of a phonologically matched word-like pseudoword and its deviants kway–kwade–kwait, was aimed at presenting the same phonological contrasts without any overt semantic or morphosyntactic information, thus controlling for phonological differences. Finally, as a further condition controlling for purely physical acoustic change, an unintelligible average of the first three (word and word-like) stimuli constituted a fourth, non-speech control condition.

The standard and deviant words for this experiment were selected mainly on the basis of their phonological characteristics. Spoken log lemma frequencies (from CELEX) for the deviant words are as follows: play(ed), 2.61; trade 2.27; plate, 1.28, trait, 0. Lexical frequency has recently been shown to affect the evoked deviant response (Alexandrov, Boricheva, Pulvermüller, & Shtyrov, 2011; Shtyrov, Kimppa, Pulvermüller, & Kujala, 2011), and it would therefore have been preferable to select deviant items more perfectly matched for frequency. The pairs of deviant words for each ending, however (i.e., played–trade and plate–trait), fall within relatively similar frequency ranges. The potential effects of lexical frequency are further considered in the Results. The vital point is that, despite some variations in lexical frequency, the stimuli were tightly controlled for acoustic and phonetic factors to ensure that all mismatch responses were elicited by physically identical contrasts.

To generate the stimulus materials, a large set of words, including multiple tokens of each spoken standard (play, tray and kway), were digitally recorded (sampling rate 44.1 kHz) by a female native speaker of British English in a soundproof room. From these materials, exemplars of each play, tray and kway were selected that were maximally similar acoustically, as each spoken token had the same fundamental frequency (*F*_0_) and duration. To avoid differential co-articulation cues that could vary with the onset of acoustic deviance and thus aid stimulus recognition within the deviant stimuli, similar spoken tokens that were not among the experimental stimuli but contain the target /d/ and /t/ endings (hade and hate) were also recorded; /d/ and /t/ endings taken from these words were appended to the end of each standard stimulus to generate the deviant stimuli. This way, all standard–deviant contrasts were identical, and the deviant stimuli could only be recognised at the last stop consonant. Thus, the deviant stimuli only diverged within their surrounding contexts, which permitted brain responses be time-locked to this precise point.

To ensure that the generated set of real-word deviant stimuli could be correctly identified by participants as the desired target tokens, we behaviourally pre-tested a range of deviant exemplars of the two real-word conditions (play and tray), created by combining the /d/ and /t/ endings with different lengths of the two standards (ranging from 290 ms to 320 ms) and varying the closure periods (ranging from 5 ms to 20 ms for the /d/ ending and 80–95 ms for the /t/ ending). Naturally spoken tokens (e.g., played, plate, trade, trait) were used to provide a direct comparison to the generated deviant stimuli. A total of 40 deviant stimuli were generated (consisting of 10 played, 10 plate, 10 trade, 10 trait stimuli) and included in the pre-testing. Eighteen participants, none of whom took part in the MEG experiment, listened via headphones to spoken tokens randomly presented on a computer. Each participant was asked to identify each word they heard and to rate how natural they thought the spoken token was on a scale of 1 to 5 (1=not at all to 5=natural). Each token was heard only once.

The four naturally spoken deviant tokens were correctly identified in every instance. The average ratings for the natural deviant tokens ending in /d/ were 4.78 for played and 3.61 for trade. From the generated deviant materials, we selected the specific deviant stimuli that were rated as most natural relative to the naturally spoken tokens. The average ratings for the generated deviant tokens ending in /d/ that were employed in the study were 3.83 for played and 4.28 for trade. These tokens of played and trade were successfully identified by all 18 pre-test participants and were not significantly different from one another in terms of the group average rating (*t*(34)=−1.73, *p*=0.09. The selected played and trade stimuli had a 310 ms stem length and the onset of the /d/ ending started 10 ms after the end of the stem.

The average ratings for the natural deviant tokens ending in /t/ were 4.72 for both plate and trait. The ratings for the generated deviant tokens selected for the experiment were 3.69 for plate and 3.31 for trait. The plate and trait stimuli generated in this manner were each correctly identified by 16/18 participants. Again, the average group ratings of naturalness were not significantly different between tokens (*t*(30)=1.08, *p*=0.29). These selected plate and trait items were again matched for stem duration and the /t/ ending began 90 ms after the end of the stem.

Although the generated pseudoword deviants were not pretested, the selected stimuli for this condition had the same fundamental frequency, duration and closure period as the selected real-word deviants. All of the selected stimuli were normalised to have the same loudness by matching root-mean-square (RMS) power across conditions (see Fig. 1).

### 2.3 Acoustic stimulation

For each condition, standards and the two associated deviant stimuli were presented within a single run of approximately 17 min duration and comprising 1000 stimuli. The inter-stimulus interval was 1000 ms. Stimuli were presented binaurally via earpieces connected to an E-Prime setup (Psychology Software Tools, Pittsburgh, PA; www.psnet.com). In each condition, each deviant stimulus (e.g., played or plate) was presented with a 10% probability among the repetitive standard stimuli. After four consecutive standard stimuli, the fifth stimulus was a deviant, with a random but equal probability of it ending with a /d/ or /t/. Presentation of conditions was counterbalanced across participants.

### 2.4 Magentoencephalographic recording

The participants were seated upright in a magnetically shielded room and instructed to focus upon watching a silent movie and pay no attention to the auditory stimuli. The evoked magnetic field responses to the stimuli were recorded (passband 0.03–200 Hz, sampling rate 1000 Hz) with a whole-head 306-channel MEG set-up (Elekta Neuromag, Helsinki) during the auditory stimulation. For offline artefact rejection, bipolar electro-oculogram (EOG) was recorded through electrodes placed above and below the left eye (vertical) and at the outer canthi of each eye (horizontal).

### 2.5 Data processing

The raw data were subjected to offline noise cancellation methods using spatiotemporal signal space separation technique (tSSS, Taulu & Kajola, 2005) implemented in MaxFilter software (Elekta Neuromag) and downsampled by a factor of 3. Data were then reduced to 65 principle components with independent components (ICs) identified using an extended version of the Independent Components Analysis (ICA) approach (using the EEGLAB toolbox, UCSD: http://sccn.ucsd.edu/eeglab). ICs that correlated maximally with the bipolar EOG recordings were isolated and removed. All subsequent preprocessing was conducted in SPM5 (http://www.fil.ion.ac.uk/spm). Following the ICA pre-processing, the data were filtered (bandpass 0.5–44 Hz) and epoched from −50 ms to 850 ms, with baseline-correction relative to the 50 ms period prior to onset of the deviant endings. That is, the event-related fields (ERFs) were resynchronised separately for the /d/ and the /t/ deviants, such that 0 ms corresponded to the onset of the ending (/d/ or /t/), where the stimuli could be uniquely identified. Epochs in which the signal from any gradiometer channel exceeded 5000 ft/cm were excluded.

The evoked difference responses for each condition were calculated by subtracting the averaged standard response from each accompanying deviant response (i.e., played minus play; plate minus play and so on). To quantify the event-related magnetic fields, vector sums of recordings in the maximally responsive planar gradiometer pairs, located over the left (0242/3) and right (1322/3) temporal lobes, were computed and the resulting vector’s absolute magnitude was used in further analyses. Previous studies have also found the maximally responsive channel in the right hemisphere to be slightly more anterior than the left, consistent with known anatomical variations between hemispheres (Alho et al., 1998; Pulvermüller et al., 2001). In addition, the average variance in the signal-to-noise ratio (SNR) was calculated across standards and deviant conditions and ending type. Mean SNR confirmed that the selected gradiometer pairs provided the maximal SNR compared to a group of 8 sensors clustered around the selected gradiometer pairs in each hemisphere. The SNRs at the maximally responsive channels in the left and right hemispheres, respectively, were 7.18 (compared to 6.2 for the clustered group on the left) and 5.9 (compared to 5.63 for the clustered group on the right).

Given the improved SNR for the two sensors, further analyses were restricted to these gradiometer pairs. Average power across a 40 ms time window around the peak in the grand-mean response was then computed and compared across conditions and hemispheres. In addition to analysis of magnitude, we quantified the hemispheric asymmetry of brain responses using a laterality quotient (*Q*):

$$Q=\frac{S_{l}-S_{r}}{S_{l}+S_{r}}\times 100$$

where the response in *S*_*l*_ and *S*_*r*_ are response magnitudes from the selected left and right hemisphere channels, respectively.

## 3 Results

**Fig. 2:**
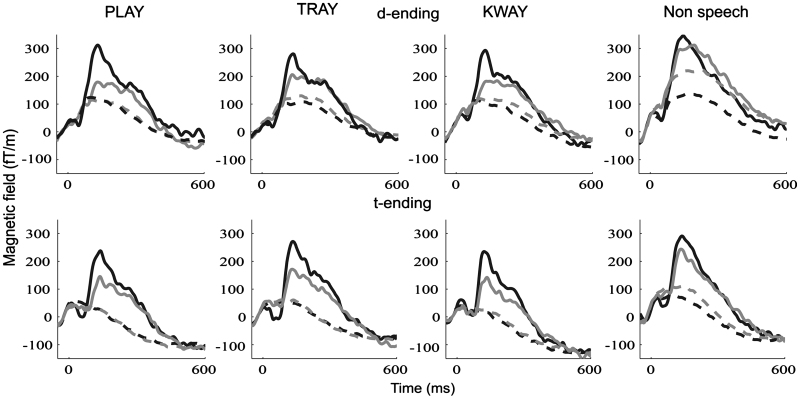
Standard and deviant response plotted for each condition and ending type. Solid lines indicate deviant responses and dashed lines indicate standard responses. Black lines indicate left hemisphere responses and pale grey lines indicate right hemisphere responses.

Event-related fields were successfully recorded and difference responses were calculated for all four conditions in both hemispheres. For an initial view of the data, the responses at the maximally responsive channels (as explained in Section 2) over a 600 ms time window are plotted separately for each of the four conditions (the columns in Fig. 2), for each of the two endings (the rows in Fig. 2), for the standard and deviant stimuli (dotted vs. solid lines) and for the two hemispheres (black vs. grey lines). Consider first the three spoken word/word-like conditions (play, tray and kway): the most notable pattern to emerge is that, although the magnitudes of the responses to the *standard* stimuli (dotted lines) were virtually identical at sensors over the left and right hemispheres, the marked responses to the *deviant* stimuli (solid lines) were always larger over the left than the right sensor. In the non-speech condition, the profile of the response to the standard and the deviant stimuli over the left hemisphere was similar to that seen for the word conditions. Over the right hemisphere, the magnitude of the response to the deviant stimulus was also akin to deviant responses in the word conditions. In contrast, the standard non-speech stimulus evoked an elevated response in the right relative to the left sensor.

Statistical analysis of the standard data using a repeated measures ANOVA with hemisphere (left, right) and condition (play, tray, kway and non-speech) as factors confirmed a significant interaction between hemisphere and condition (*F*(3,51)=4.33, *p*=0.01) with a strong trend towards a larger right hemisphere response for non-speech (*t*(35)=1.89, *p*=0.07), but not for the remaining three conditions (all *t*-values <1). Analysis of the deviant data, which included ending (/d/ vs. /t/) as an additional factor, provided a complementary pattern. The hemisphere by condition interaction was not significant (*F*(3,51)=2.17, *p*=0.1), but non-parametric *t*-tests, adjusted for multiple comparisons, indicated that the three word or word-like conditions all elicited a differentially larger left>right sensor response (deviants: play: *t*(35)=3.82, *p*=0.001; tray: *t*(35)=3.33, *p*=0.002; kway: *t*(35)=3.39, *p*=0.002) relative to the non-speech condition (*p*=0.2).

**Fig. 3:**
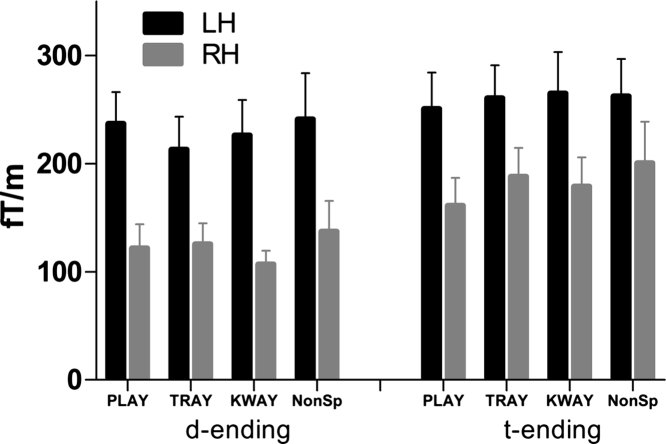
Averaged vector sum data from a 40 ms time window at peak of the difference response in maximally responsive channels in the left and right hemisphere. NonSp refers to non-speech.

Next a standard–deviant difference (MMN) wave was calculated for each condition and the analysis was restricted to the mean amplitude data across a 40 ms time window ranging from 110 ms to 150 ms. The peak around which the 40 ms time window was placed was identified by averaging the grand mean responses of each condition. A repeated measures ANOVA with hemisphere (left, right), condition (play, tray, kway and non-speech) and ending type (/d/ and /t/) as factors indicated a bilateral but markedly left>right response (*F*(1,17)=9.27, *p*<0.01) (see Fig. 3). Post hoc *t*-test comparisons revealed that responses were reliably stronger on the left for every condition, except non-speech appended with a *t*-ending (*t*(17)=1.63, *p*=0.12). There were no reliable differences between the magnitudes of the responses to each condition (*F*(3,51)=0.73, *p*=0.54), suggesting that any variation in the evoked response across conditions was not simply due to minimal variations in lexical frequency. Rather, a significantly larger difference occurred in response to the /t/ compared to the /d/ endings (*F*(1,17)=14.12, *p*<0.01). A significant interaction was observed between ending type and hemisphere (*F*(1,17)=5.4, *p*=0.03), with the sensor over the right hemisphere responding more strongly to the /t/ ending. There were no other significant interactions.

**Fig. 4:**
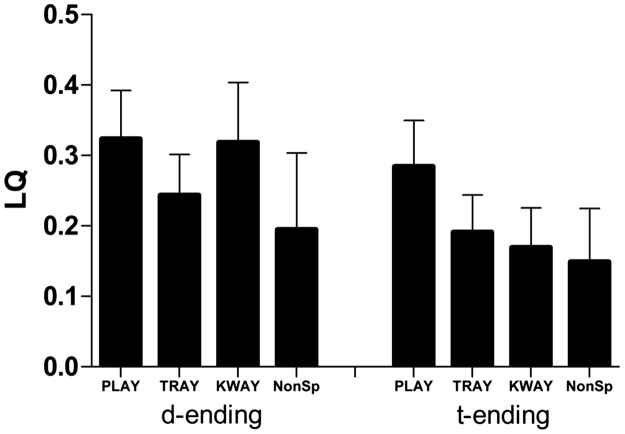
Laterality quotient data averaged across a 40 ms time window for each condition and ending type. NonSp refers to non-speech.

These laterality differences between conditions and ending types were scrutinised in the further analyses. A laterality quotient was calculated for each sample point in an epoch and statistical analyses performed on data averaged across the 40 ms window at the peak of the response. All laterality quotients were greater than zero at this early peak around 130 ms, once again indicating a larger left hemisphere response across all conditions (see Fig. 4).

Focusing on the /d/ ending in Fig. 4, strong left laterality of the difference response is apparent for the conditions played and kwade/kwayed (mean LQ for both=0.32), but less so for trade (0.24) and the non-speech (0.19) condition. A Wilcoxon signed rank test confirmed that played and kwade/kwayed were not significantly different from one another (*Z*=−0.15, *p*=0.88). The laterality of the difference response to played showed a significant left lateralisation when compared to trade (*Z*=−1.851, *p*=0.03, one-tailed), and non-speech (*Z*=−1.894, *p*=0.03, one-tailed). Likewise, kwade/kwayed was significantly more left lateralised than non-speech (*Z*=−2.11, *p*=0.04). Direct comparison to trade showed a strong trend towards kwade/kwayed being more left lateralised (*Z*=−1.50, *p*=0.06, one-tailed). There was no significant difference between trade and non-speech (*Z*=−0.65, *p*=0.95). One potential explanation for this variation is that trade, despite sounding as if it might have an affixed ending (i.e., what Marslen-Wilson & Tyler, 2007, call an inflectional rhyme pattern), is also a known whole monomorphemic word, unlike played (which is only an affixed word) and kwade (which is not a known word but, if it were, might be affixed: “kwayed”). To explore this suggestion further, average laterality quotients computed across this 40 ms time window for played and kwade were collapsed and directly compared to trade and the non-speech condition. A Friedman test performed on averaged data from this interval indicated a significant difference between conditions that ended in a plausible affix (played and kwade) compared to the other two conditions (*χ*^2^=7.8, *df*=3, *p*=0.05). This pattern is specific to the /d/ ending and was not observed in the /t/ conditions (*p*=0.3).

## 4 Discussion

The specific purpose of this study was to analyse early magnetic brain responses to three sets of contrasting forms of deviant stimuli in a passive oddball paradigm. Across the four conditions for trials where the deviant stimuli ended in /d/, the first contrast was between the two deviants that are actually or plausibly affixed (played and kwayed) vs. those unlikely to be treated in that fashion (trade because it is a known monomorphemic word and the non-speech deviant because it is not a recognisable word or pseudoword). The second more spüecific contrast was between the two real-word deviants consisting of an inflected regular English verb (played) vs. its phonological twin (trade). Finally, across all conditions, the third contrast was between deviants ending in /d/ vs. /t/. The measures employed to address these issues were the amplitude of the brain responses and their likely left vs. right-hemisphere origins as reflected in responses recorded by channels over the left and right temporal lobes.

Before we discuss the information provided by this study regarding these issues, it is worth a brief reminder as to the basis for their interest. The first contrast derives its particular motivation from the neurobiological model (Pulvermüller, 1995, 2005) according to which affixes, be they attached to real meaningful words or to pseudowords (played and kwayed), are expected to generate stronger left-hemisphere responses than monomorphemic words (trade) or non-speech deviants. As discussed in the Introduction, this predicted pattern is consistent with separate mechanisms for regular and irregular verbs (as postulated Pinker & Ullman and Tyler & Marslen-Wilson), since irregular past-tense forms do not have explicit affixes. On the other hand, this prediction of differential laterality in fact derives from the well-founded assumption that phonology is left lateralised whereas semantic networks are bilateral. The actual explanation in this neurobiological framework, therefore, relies on the particular combination of phonological vs. semantic processes recruited in the analysis of regular past-tense verbs and their phonological twins; this is consistent with the proposal (as argued by McClelland and Patterson (2002)) that regular and irregular verbs do not require separate mechanisms.

The motivation for the second and third contrasts concerns previous neuropsychological findings. Two studies of past-tense processing by patients with non-fluent aphasia (Bird et al., 2003; Tyler et al., 2002c) documented roughly equal impairment in the patients’ ability to judge spoken pairs of words as different when the pairs consisted of either stem and inflected forms like “play–played” or phonological twins like “tray–trade”. Tyler et al. (2002c), measuring response times as well as accuracy, concluded that the patients’ main deficit was in morphological processing of words like “played”. This was because – when the patients did make correct different judgements in the morphological and phonological-twin conditions – their responses were slower to the pairs containing true inflections. Bird et al. (2003), measuring only accuracy, concluded that the patients’ main deficit was in phonological processing of words like “played”. This was partly because of the patients’ virtually identical accuracy for morphological pairs and their phonological twins, but also because the experiment included an additional condition in which one member of the pair ended in two phonemes with discrepant voicing (such as “play–plate”), which cannot occur in regular verb stems and their inflected forms. This phonological factor of consistent vs. inconsistent voicing was a major determinant of the patients’ success rate in different judgements, independent of morphological status of the words. These neuropsychological findings form the basis for our particular interest in the second and third contrasts specified above: played vs. trade and /d/ vs. /t/ endings. The current study, in healthy participants rather than patients, measured automatic magnetic responses of the brain immediately post stimulus presentation. This more sophisticated and subtle paradigm offers a fruitful and complementary avenue to addressing these research questions.

Regarding the first main question, whether responses to real and plausibly affixed deviants are more left lateralised than those to monomorphemic and non-speech deviants, the answer is yes (see the d-ending panel of Fig. 4). This is an important result because it demonstrates that strictly behavioural measures on this topic – from people with healthy brains or even from patients with brain lesions – are insufficient to reveal how stems and affixes are processed by the brain. As already mentioned, neither the neurobiological theory underlying this prediction nor our result supporting the theory enables a clear decision between dual- and single-mechanism approaches to past-tense verb processing. The stronger left>right hemisphere laterality quotients observed for the played and kwayed deviants seems most consistent with the proposal of a special-purpose, rule-based, left-hemisphere process for regular verbs, as specified in dual-mechanism theories; but the result is also compatible with the single-mechanism account, especially given the emphasis in this account on a greater role for left-hemisphere phonology in processing regular past-tense forms. Note also that the left>right laterality quotients for played and kwayed (Fig. 4) were significantly, but not massively, larger than the quotient for trade. To obtain more definitive MEG evidence on this question, one would need more extensive stimulus conditions, including not only regular past-tense items but also both irregular past-tense words without frank inflections (such as ran or bought) and ‘pseudo-inflected’ irregular past tense forms (such as slept). The important point is that only a neurophysiological technique like MEG can provide such evidence.

In an fMRI study, Marslen-Wilson and Tyler (2007) reported greater left laterality for words and pseudowords with an inflectional rhyme pattern, and interpreted this result as reflecting a highly automatic decomposition triggered by morpho-phonological features. The general outcome of our study, which investigated automatic brain responses to stimuli outside the focus of attention, is consistent with their position, but the precise pattern is not in that the monomorphemic item trade did not elicit as strong laterality as played and kwade/kwayed, thus not supporting a strong contribution of morphological rhyme pattern. Our data suggest that, even if there is rapid online decomposition of a word such as trade into tray-ed, recognition of the whole word trade automatically invokes bilateral semantic circuits, thus possibly overriding any competing affixation or decomposition processes. The observed pattern of differential laterality seems best explained by the greater morpho-phonological demand placed on the left-lateralised perisylvian language system by affixed words, whereas the trend towards more symmetric responses to whole monomorphemic forms is consistent with the need to bind these word forms with meaning, a more widely distributed and bi-hemispherically more balanced mechanism (Patterson, Nestor, & Rogers, 2007; Pulvermüller, 1999).

The second main question is a sort of refinement of the first: specifically, does the brain respond differently to an inflected deviant real word vs. its real word phonological twin? The answer to this is that it depends on which measure one attends to. As assessed by amplitude of the deviant response (see play vs. tray with /d/ endings in Fig. 2), the answer emerging from the present results is no, or – since one cannot accept a hypothesis on the basis of lack of evidence – at least not definitively yes. On the other hand, as assessed by laterality quotient, the answer from the present data is yes, with a larger left>right pattern for the affixed word played than for the monomorphemic word trade (Fig. 4). This is a potentially exciting outcome: even though we cannot claim any direct correspondence between the different MEG measures (laterality vs. amplitude) and the different behavioural measures used in neuropsychological studies (response times vs. accuracy), at least we have demonstrated that one MEG measure discriminates between brain responses to played vs. trade and one does not, just as one behavioural measure apparently does and one does not.

Regarding the third main question – whether the brain responds differently to deviants with consistent voicing (like played, trade and kwade) vs. those ending in inconsistently voiced phonemes (plate, trait and kwate) – the answer is yes: the evoked difference responses between deviant and standard were larger for the inconsistently voiced /t/-ending deviants, especially over the right hemisphere (Fig. 3). This is also an exciting result, because of the large advantage observed by Bird et al. (2003) in patients’ judgements about inconsistently voiced words. The nonfluent aphasic patients in that study were considerably more accurate in making ‘different’ judgements to word pairs including discrepant voicing, like “he–heat”, “heed–heat” and “an–ant” (accuracy>80%), than to pairs with only consistent voicing like “he–heed”, “an–and”, “chess–chest” or “press–pressed” (accuracy in the range 38–58%). Such patients have large left-hemisphere lesions but intact right hemispheres, and the greater MEG difference response in the right hemisphere to plate, trait and kwate than to played, trade and kwade in healthy participants is therefore consonant with, and may even explain, the patient data.

One possible explanation for the main effect of larger responses to /t/ than /d/ endings is that the /t/ ending is acoustically more salient, because there was a longer closure period between the offset of the stem and the onset of the burst for /t/ than for /d/ (90 ms vs. 10 ms). In addition, the plosion of the /t/ sound had higher sound energy and lasted longer than that of the /d/ (see Fig. 1). An explanation for the interaction between final phoneme (ending type) and hemisphere, on the other hand, may also involve linguistic factors, for example the voicing contrast between the phonemes /d/ and /t/ or the morphological and semantic status of the deviant stimulus. Whatever the relative contributions of the two hemispheres to acoustic processing, there can be no doubt of a bigger contribution of the language-dominant left to specifically linguistic processes such as morphology and phonology. Note that these two explanations – saliency and energy vs. linguistic factors – are not mutually exclusive, and indeed it is plausible that both factors contributed to the results.

Finally, although not one of the specific motivations for this study, we confirmed left-lateralised responses for all three spoken word and word-like conditions, as have previously been reported in EEG and MEG (Kujala, Alho, Service, Ilmoniemi, & Connolly, 2004; Shtyrov et al., 2000; Shtyrov, Pihko, & Pulvermüller, 2005) and PET/fMRI studies of language processing (e.g., Alho et al., 2003; Price, 2000; Tervaniemi & Hugdahl, 2003). The timing of the evoked mismatch response reported in the present study is also consistent with the N1m (the magnetic counterpart of the N1) response indexing acoustic analysis (Näätänen & Picton, 1987), seen in other studies that use a fully random oddball design, and the mismatch negativity (MMN) response indexing auditory discrimination as well as activation of linguistic long-term memory traces (Shtyrov & Pulvermüller, 2007).

One slight puzzle in our findings is the enhanced right hemisphere activation for the non-speech stimuli. Previous experiments with non-speech stimuli using passive oddball designs have also suggested a tendency for right-hemispheric preponderance of mismatch responses (e.g., Paavilainen, Alho, Reinikainen, Sams, & Näätänen, 1991). Our non-speech sounds were of a more complex nature than those used in earlier experiments. Still, given that the non-speech stimuli were generated by averaging all word/wordlike conditions together, the prosodic characteristics that typified the word conditions may well have been partially preserved, and the presence of prosody in the absence of clearly identifiable phonetic/phonemic content may recruit right hemisphere involvement (Belin, Zatorre, Hoge, Evans, & Pike, 1999; Belin, Zatorre, Lafaille, Ahad, & Pike, 2000). Indeed, in an MEG study exploring degraded relative to original speech sounds, the degraded stimuli increased the amplitude of the evoked response in the right hemisphere N1m (Liikkanen et al., 2007).

In summary, we have demonstrated that rapid online neuromagnetic responses to monomorphemic words, affixed words, pseudowords and non-speech stimuli are influenced by acoustic, phonological, morphological and semantic factors. The results offer considerable insight into previously rather mystifying, and sometimes conflicting, outcomes of purely behavioural, neuropsychological studies.

## Figures and Tables

**Table 1:** Auditory stimuli used in the four experimental conditions. All stimuli were maximally matched for their acoustic properties (cf. Fig. 1). The standard–deviant contrasts of interest are equivalent across all four conditions.

	**PLAY**	**TRAY**	**KWAY**	**Non-speech**
Standard	PLAY	TRAY	KWAY	NON-SPEECH
Deviant +/d/ ending	PLAYED	TRADE	KWADE	NON-SPEECH+/d/
Deviant +/t/ ending	PLATE	TRAIT	KWATE	NON-SPEECH +/t/
